# Diurnal variation of tension-type headache intensity and exacerbation: An investigation using computerized ecological momentary assessment

**DOI:** 10.1186/1751-0759-6-18

**Published:** 2012-09-04

**Authors:** Hiroe Kikuchi, Kazuhiro Yoshiuchi, Yoshiharu Yamamoto, Gen Komaki, Akira Akabayashi

**Affiliations:** 1 Department of Psychosomatic Research, National Institute of Mental Health, National Center of Neurology and Psychiatry, 4-1-1 Ogawa-Higashi, Kodaira, Tokyo, 187-8553, Japan; 2 Department of Stress Sciences and Psychosomatic Medicine, Graduate School of Medicine, The University of Tokyo, 7-3-1 Hongo, Bunkyo-ku, Tokyo, 113-8655, Japan; 3 Educational Physiology Laboratory, Graduate School of Education, The University of Tokyo, 7-3-1 Hongo, Bunkyo-ku, Tokyo, 113-0033, Japan

**Keywords:** Tension-type headache, Ecological momentary assessment, Electronic diary, Diurnal variation

## Abstract

**Backgrounds:**

Tension-type headache is a common psychosomatic disease. However, diurnal variation of headache is yet to be clarified, perhaps due to the lack of an appropriate method to investigate it. Like other painful diseases, it would be helpful to know if there is diurnal variation in tension-type headaches, both for managing headaches and understanding their pathophysiology. The aim of this study was to determine if there is diurnal variation in the intensity and exacerbation of tension-type headache.

**Methods:**

Patients (N = 31) with tension-type headache recorded for one week their momentary headache intensity several times a day and their acute headache exacerbations using a watch-type computer as an electronic diary (computerized ecological momentary assessment). Multilevel modeling was used to test the effects of time of day on momentary headache intensity and on the occurrence of acute exacerbations.

**Results:**

A significant diurnal variation in momentary headache intensity was shown (*P* = 0.0005), with the weakest headaches in the morning and a peak in the late afternoon. A between-individual difference in the diurnal pattern was suggested. On-demand medication use was associated with a different diurnal pattern (*P* = 0.025), suggesting that headache intensity decreases earlier in the evening in subjects who used on-demand medication, while headache subtype, prophylactic medication use, and sex were not associated with the difference. The occurrence of acute headache exacerbation also showed a significant diurnal variation, with a peak after noon (*P* = 0.0015).

**Conclusions:**

Tension-type headache was shown to have a significant diurnal variation. The relation to pathophysiology and psychosocial aspects needs to be further explored.

## Backgrounds

Tension-type headache (TTH) is one of the common and major diseases in psychosomatic medicine because of its high prevalence and supposed association with psychosocial factors. Despite its familiarity, diurnal variation of headache, which is thought to be a quite basic characteristic, is still unclear. One of the reasons was lack of an appropriate method to investigate a detailed diurnal variation. One cross-sectional study reported that almost half of the TTH patients had their usual onset of headache attacks during the day [1]. However, this report relied on the patients’ recalled perception regarding what time their headaches most frequently had occurred. It has often been pointed out that recall bias is inevitable in surveys based on subjects’ recall. No prospective study has been conducted to confirm temporal distribution of TTH attacks throughout the day. Moreover, diurnal variation of headache intensity in TTH has rarely been investigated, especially by prospective studies.

Recently, ecological momentary assessment (EMA), which avoids recall bias, has been used in the area of psychosomatic medicine to document subjective symptoms in subjects' daily lives [2]. EMA is a method to assess and record symptoms at the moment they happen via recording throughout their daily lives. It is done using paper-and-pencil diaries or electronic diaries (i.e. computerized EMA). In TTH, consistency between recalled headache intensity and momentary headache intensity recorded by computerized EMA was shown to be low in our previous study [3]. In addition, compared with EMA using paper-and-pencil diaries, computerized EMA has the advantage of avoiding 'faked compliance', i.e. disguised compliance by recording data at times other than those designated, by recording the time of data input [4]. This feature is particularly appropriate for investigating temporal symptom patterns.

Diurnal variation of pain intensity has been discussed in several types of pain such as cancer and arthritis pain in the context of optimization of pain control and the relation to pathophysiology [5,6]. In TTH as well, investigation of diurnal variation of headache intensity would be helpful for patient education, headache management, and the understanding of their pathophysiology. Therefore, the aims of this study were to prospectively investigate the diurnal pattern of TTH in the daily setting using computerized EMA and to determine if there is diurnal variation of headache intensity in TTH and if there is a specific time of day when acute headache exacerbations are more likely to occur.

## Methods

All procedures and materials were approved by the ethics committee of the University of Tokyo.

### Subjects

The subjects were participants in a trial of relaxation therapy for TTH and overlapped with the subjects in a previously reported study [7]. Recruitment was by an advertisement on websites from March 2003 to August 2004. Patients who applied were interviewed and screened by well-trained physicians.

Inclusion criteria included age 20 years or older but less than 60 years with any type of TTH according to the criteria of the International Headache Society [8] and with at least one headache episode per week, on average. The patients were excluded if they had headache other than TTH according to the criteria of the International Headache Society [8]; current psychiatric disease; history of paranoia or schizophrenia; severe physical illnesses; if they had participated in relaxation therapy; and if they were employed as a shift worker. Diagnosis of psychiatric disease was made according to the criteria in the Diagnostic and Statistical Manual of Mental Disorders, Fourth Edition, Text Revision [9].

Eighty-four subjects applied to participate in the study. Ultimately, 54 of 59 eligible subjects were enrolled in the relaxation therapy trial; Five declined to participate due to scheduling conflicts. After the exclusion of 19 subjects with migraine and one subject who worked as a shift worker, 34 remained eligible for study. All the subjects gave their written informed consent to participate.

### Measurement of momentary headache intensity

Momentary headache intensity was recorded with watch-type computers (Ruputer ECOLOG; 42 grams, Seiko Instruments Inc., Tokyo, Japan) used as electronic diaries [3,7]. The computer had a screen measuring 20 × 30 mm and a joystick and button as input devices. The subjects were fully instructed how to use the device and given manuals before the beginning of the study period. They also practiced manipulating the device with one of the authors (HK) until they became accustomed to its use.

The subjects wore the watch-type computers for seven consecutive days before relaxation therapy started. Signal-contingent recordings, which were defined as recordings prompted by a beep [2], were programmed to occur randomly within an interval of 36 minutes at approximately 6:00, 12:00, 18:00, and 24:00. The subjects were allowed to postpone input for 30 minutes. Recordings not made within 30 minutes were cancelled. The subjects were also asked to record their headache intensities when waking up and going to bed by choosing ‘waking up’ or ‘going to bed’ from the menu. After selecting ‘going to bed’, computers suspended the beeps for signal-contingent recordings until a ‘waking up’ recording was selected to avoid disturbing the subject’s sleep. Signal-contingent recordings and recordings when waking up and going to bed were treated as scheduled recordings.

Event-contingent recordings were defined as recordings that were initiated by the subjects themselves when a particular event occurred [2]. In this study, the subjects were asked to make a recording as soon as possible every time their headache became exacerbated as an event-contingent recording.

In both scheduled and event-contingent recordings, momentary headache intensity was rated according to a visual analog scale (VAS) from 0 to 100 displayed on the screen. The words "headache intensity" were displayed with the VAS as a question, and the anchor words "none" and "most intense" were displayed at the respective ends of the scale. The subjects manipulated the joystick and adjusted the length of the bar so that it corresponded to their headache intensity at that moment.

### Statistical analysis

Multilevel modeling was used to investigate diurnal variation of headache intensity and acute headache exacerbations, using SAS Proc Mixed and Proc Nlmixed (SAS 9.1, SAS Institute Inc., Cary, NC).

First, time of day was divided into three-hour blocks (e.g. 0:00-3:00, 3:00-6:00, etc.) and treated as a categorical variable. Momentary headache intensity was treated as the dependent variable and time of day as the predictor. The effect of time was modeled either as fixed or random. Therefore, the following models were tested: model A1, an unconditional model (no predictor); model A2, a model with the effect of time as a fixed effect; model A3, a model with the effect of time as a random effect. The level 1 intercept was the individual true value of the momentary headache intensity when the time of day was in the reference block (21:00-24:00), and it was modeled as a random effect. The variance-covariance matrix (G matrix) was modeled as variance component structure.

Second, the effect of time on the logarithm of the average number of headache exacerbations in three hours was tested by using a multilevel Poisson model in order to investigate what time of day headache exacerbations were most likely to occur. The number of headache exacerbations for every three hours were calculated for each patient as the outcome variables, and time of day was divided into three-hour blocks as described above as a categorical variable. The time block 3:00-6:00 was excluded from the analysis because no subjects had headache exacerbation during that time block. Effects of time were modeled as fixed. The following models were tested: model B1, an unconditional model (no predictor); model B2, a model with the effect of time (three-hour blocks) as a fixed effect. The level 1 intercept (individual true value of the logarithm of the average number of headache exacerbations when the time of day was in the reference block (21:00-24:00)) was modeled as a random effect.

Third, we investigated the difference of the diurnal pattern between chronic and episodic tension-type headache by analyzing the models with a variable of headache subtype and the interaction term of time and headache subtype in order to investigate the heterogeneity of diurnal pattern. Headache diagnosed as "headache of tension-type not fulfilling the criteria of episodic or chronic tension-type headache" was coded as either chronic or episodic according to average headache frequency (≥ 15 days/month or not). We also investigated the effect of medication use on the diurnal variation of TTH by analyzing the models with a dichotomous variable of medication use and the interaction term of time and medication use. We tested two types of medication use: The use of prophylactic medication and the use of on-demand analgesic medication during the study period. In addition, we investigated the difference of the diurnal pattern between women and men.

Goodness of fit was compared using a -2 log likelihood function and *χ*^2^ test when one model was nested in the other; otherwise Akaike’s Information Criterion (AIC) was used. Post-hoc multiple comparisons were conducted with either Turkey-Kramer correction or Bonferroni correction. The significance level was set at 0.05.

## Results

### Patient characteristics

Thirty-four subjects were enrolled in the study and three were excluded from further analysis because they were unable to complete their recordings for seven days due to problems with the computers. Finally, data from 31 subjects (nine men and 22 women) were analyzed. The profiles of all subjects are shown in Table 1, with women and men listed separately. The mean age of the subjects was 38.4 years (SD, 10.4 years; range, 25-60 years). Five subjects had episodic TTH, 24 had chronic TTH, and two had headache of tension-type not fulfilling the criteria of episodic or chronic tension-type headache. Thirteen subjects answered that they regularly took prophylactic medication and 19 answered that they usually used analgesics on-demand. The subjects who were excluded from the analysis were all men, and two of whom had chronic TTH and the other had TTH not fulfilling the criteria of episodic or chronic tension-type headache. Neither their ages nor headache intensities rated by weekly recall were significantly different from those of the subjects who completed their recordings.

**Table 1:** Demographic and medical characteristics of the subjects

	**Total (N = 31)**	**Women (N = 22)**	**Men (N = 9)**
Age			
Mean in years (SD)	38.4 (10.4)	38.1(11.1)	38.7(9.0)
Subtype of tension-type headache			
Episodic	5 (16%)	4	1
Chronic	24 (77%)	16	8
Other*	2 (6%)	2	0
Disease duration			
Median in years (range)	7.0 (0.1-24.0)	7.5 (0.1-24.0)	3.0 (1.3-23.0)
Prophylactic medication			
With	13 (42%)	8	5
anxiolytics	9 (29%)	5	4
muscle relaxants	5 (16%)	2	3
Chinese herbal medicine	4 (13%)	4	0
antidepressants	3 (10%)	1	2
Without	18 (58%)	14	4
Use of on-demand medication			
With	19 (61%)	15	4
analgesics (either as a single or compound preparation)	14 (45%)	10	4
anxiolytics	5 (16%)	5	0
muscle relaxants	2 (6%)	2	0
Without	12 (39%)	7	5

*SD*, standard deviation.

* headache of tension-type not fulfilling the criteria of episodic or chronic tension-type headache.

### Recording profiles

**Figure 1:**
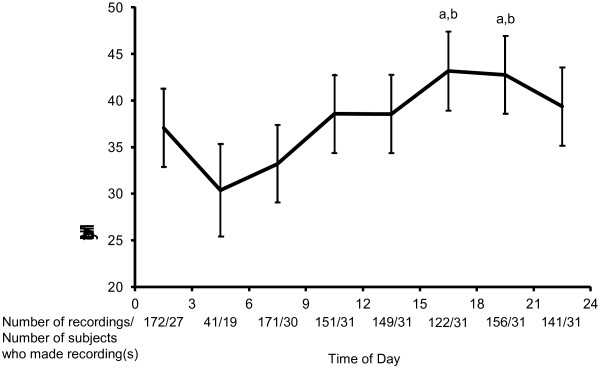
**Diurnal variation in headache intensity.** The estimated mean headache intensity is shown with its standard error for each three-hour block. a *P* < 0.05 vs. 3:00-6:00; b *P* < 0.05 vs. 6:00-9:00, using Tukey's multiple comparison.

For all subjects, there were 998 scheduled recordings, consisting of 570 signal-contingent recordings, 213 recordings on awakening, and 215 recordings at bedtime. The mean compliance rate for signal-contingent recording was 96%. The mean number of scheduled recordings was 32.6 per subject. Twenty-three subjects made 105 event-contingent recordings and the other eight subjects made no event-contingent recordings. The number of recordings made in each three-hour time block and the number of subjects who made recordings in each three-hour time block are shown in Figure 1.

### Diurnal variation of momentary headache intensity

Of the three models, the model with time as a random effect (model A3) had the best fit. The effect of time of day was significant in model A3 (F(7, 193) = 3.91, *P* = 0.0005). Multiple comparisons of all pairs of estimated momentary headache intensity from each time block (with Turkey-Kramer correction) revealed that momentary headache intensity from 3:00-6:00 was significantly lower than from 15:00-18:00 (t(193) = -3.38, adjusted *P* = 0.019) and 18:00-21:00 (t(193) = -3.35, adjusted *P* = 0.021), and that momentary headache intensity from 6:00-9:00 was significantly lower than from 15:00-18:00 (t(193) = -3.73, adjusted *P* = 0.006) and 18:00-21:00 (t(193) = -3.78, adjusted *P* = 0.0051). Model A3 is expressed as follows:

Level 1 equation:

(1) $${\text{Y}}_{\mathrm{ij}}=\pi_{0i}+\pi_{1i}\text{TIME}0_{\mathrm{ij}}+\pi_{2i}\text{TIME}3_{\mathrm{ij}}+\cdots \cdots +\pi_{7i}\text{TIME}18_{\mathrm{ij}}+\epsilon_{\mathrm{ij}}$$

Level 2 equations:

(2) $$\begin{array}{l} \pi_{0i}=\gamma_{00}+\zeta_{0i}\\ \pi_{\text{k}i}=\gamma_{\text{k}0}+\zeta_{\text{k}i}\left(\text{k}=1,2,\cdots \cdots ,7\right) \end{array}$$

where Y_*ij*_ is each momentary headache intensity for the *i*th patient; TIME0_*ij*_, TIME3_*ij*_, ······, and TIME18_*ij*_ are dummy variables indicating that the time of day was in 0:00-3:00, 3:00-6:00, ······,or 18:00-21:00, respectively (e.g. TIME0_*ij*_ = 1 if the time of day was in 0:00-3:00, otherwise TIME0_*ij*_ = 0). π_0*i*_ is the individual *i*’s true value of momentary headache intensity when time of day was in the reference block (21:00-24:00) and π_1*i*_, ······, and π_7*i*_ are individual *i*’s true differences in momentary headache intensity between corresponding time blocks and the reference time block. γs are group means of corresponding πs. ϵ_*ij*_*,* ζ_0*i*_ and ζ_k*i*_ (k = 1, 2, ······, 7) are residuals at each level and including them in the equations means that the intercept and the effect of time are modeled as random. The estimated momentary headache intensity is plotted in Figure 1.

G matrix is modeled as a variance component structure in this model, which means that ζ_k*i*_s (k = 1, 2, ······, 7) are assumed to have the same value of variance. The estimated variance of ζ_k*i*_s was 38.5, which is significantly different from zero (*P* < 0.0001).

### Average number of headache exacerbations throughout the day

The model with the effect of time (model B2) fit better than the unconditional model, and the effect of time was significant (F(6, 30) = 4.81, *P* = 0.0015). Model B2 is expressed as follows:

Level 1 equation:

(3) $$\text{Log}\left({\text{Ave}}_{\mathrm{ij}}\right)=\pi_{0i}+\pi_{1i}\text{TIME}0_{\mathrm{ij}}+\pi_{2i}{\text{TIME}6}_{\mathrm{ij}}+\cdots \cdots +\pi_{6i}{\text{TIME}18}_{\mathrm{ij}}$$

Level 2 equations:

(4) $$\begin{array}{l} \pi_{0i}=\gamma_{00}+\zeta_{0i}\\ \pi_{\text{k}i}=\gamma_{\text{k}0}\left(\text{k}=1,2,\cdots \cdots ,6\right) \end{array}$$

**Figure 2:**
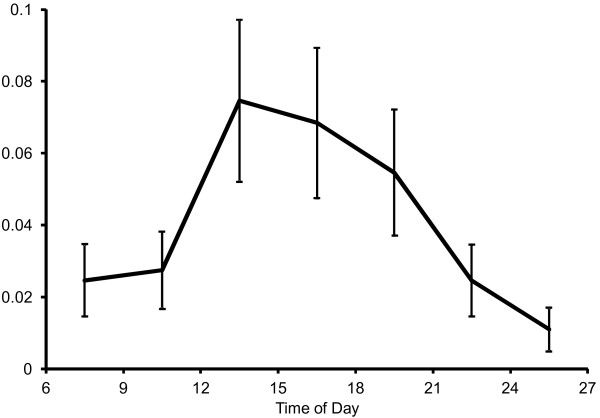
**Probability of headache exacerbation.** The estimated average number of headache exacerbations per three hours is shown with its standard error for each three-hour block.

The number of headache exacerbations in a three-hour block ~ Poisson (Ave_*ij*_)

where Ave_*ij*_ is the parameter of the Poisson distribution (average number of headache exacerbations per three hours) for each time block for the *i*th patient; and TIME0_*ij*_, TIME6_*ij*_, ······, and TIME18_*ij*_ are dummy variables for the time of day used in the same way as they are used in model A3. π_0*i*_ is the individual *i*’s logarithm of the average number of headache exacerbations when time of day was in the reference block (21:00-24:00) and π_1*i*_, ······, and π_6*i*_ are individual *i*’s differences in the logarithmic average number of headache exacerbations between corresponding time blocks, and the reference time block. γs are the group mean of corresponding πs. ζ_0*i*_ is the residual. The estimated average number of headache exacerbations per three hours is plotted in Figure 2.

Multiple comparisons of all pairs of estimated logarithmic average number of headache exacerbations for each time block (alpha level set at 0.0033 with Bonferroni correction) were conducted, and it was revealed that the logarithm of the average number of headache exacerbations from 0:00-3:00 was significantly smaller than from 12:00-15:00 (t(30) = -3.59, *P* = 0.0012) and 15:00-18:00 (t(30) = -3.41, *P* = 0.0019).

### Effect of headache subtypes on diurnal variation of momentary headache intensity and on the average number of headache exacerbations throughout the day

The models with the interaction of time and headache subtype did not fit better than the models without it. Furthermore the interaction was not significant in the models with it.

### Effect of medication use on diurnal variation of momentary headache intensity and on the average number of headache exacerbations throughout the day

The models with the interaction of time and prophylactic medication use did not fit better than those without it, either in the analyses of diurnal variation in momentary headache intensity or in the analysis of diurnal variation of headache attacks. Furthermore, the interaction of time and prophylactic medication use was not significant in the models including them.

**Figure 3:**
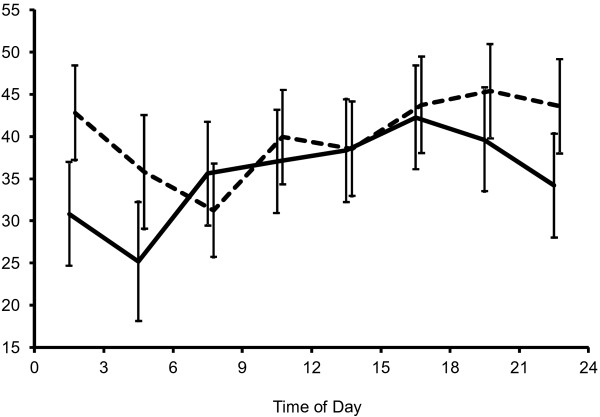
**Diurnal variation in headache intensity and on-demand analgesic medication use.** The estimated mean headache intensity is shown as a solid line for the patients who used on-demand analgesic medication during the study period and as a dashed line for those who did not, both with the standard error for each three-hour block.

The model with the effect of time as a random effect and the interaction of time and on-demand analgesic medication use fit better than the model without the interaction, and the interaction was significant (F(7,186) = 2.36, *P* = 0.025) in the analysis of diurnal variation of momentary headache intensity. The estimated momentary headache intensity for each group (patients with and without on-demand analgesic medication use) is plotted in Figure 3. The model with the interaction of time and on-demand analgesic medication use did not fit better than the model without them in the analysis of diurnal variation of headache attacks. Furthermore the interactions were not significant in the models with them.

### Effect of sex on diurnal variation of momentary headache intensity and on the average number of headache exacerbations throughout the day

The models with the interaction of time and sex did not fit better than those without it, either in the analyses of diurnal variation in momentary headache intensity or in the analysis of diurnal variation of headache attacks. Furthermore, the interaction of time and sex was not significant in the models including it.

## Discussion

A significant diurnal variation in headache intensity was shown in TTH through utilization of computerized EMA, with headache being weakest in the morning, worsening toward the evening, and peaking afterward.

When the effect of time was modeled as a random effect, the models fit better than when the effect was modeled as a fixed effect. In addition, the estimated variances of those random effects were significantly different from zero. These results suggested that there was significant between-individual variation in the effect of time. In other words, the pattern of diurnal variation of TTH was different among patients, while a certain diurnal pattern was still observed when averaged for all patients.

The heterogeneity of diurnal patterns of headache intensity may stem from different subtypes of tension-type headache and sex. However, the analysis failed to show a significant difference in diurnal patterns between chronic and episodic tension-type headache or between women and men. Another possibility is that medication use influenced the time course of headache. While prophylactic medication use did not show any significant difference in the diurnal pattern of headache intensity, on-demand medication use was related to a significant difference in diurnal pattern of headache intensity. The headache intensity decreased earlier in the evening in patients who used on-demand medication during the study period, which might reflect the effect of the medication.

The circadian variation in the probability of acute headache exacerbations also showed a specific diurnal pattern. The estimated average number of headache exacerbations was small in the morning, reached a peak just after noon, and then gradually decreased towards night. These results were consistent with a previous study in which almost half of TTH patients reported that their usual headache attack onset was during the day [1]. In this prospective study using computerized EMA, it was possible to determine more accurate and detailed information on the temporal distribution of headache exacerbations. Neither subtype of headache nor medication use was related to a significant difference of circadian variation on the probability of acute headache exacerbation.

Circadian pain intensity variation generally has been discussed in relation to endogenous opioid peptides and melatonin [5,6]. However, their involvement in TTH has not yet been established [10,11]. Furthermore, a previous study reported that the diurnal pattern itself was different for plasma melatonin between patients with chronic TTH and healthy controls [12]. Further investigation is necessary in order to clarify the biological origin of the circadian variation of pain intensity in TTH. In addition, psychosocial factors such as daily social activities and related psychological symptoms also provide a possible basis for the circadian variation in TTH. The temporal relationship between headache intensity and psychosocial factors also needs to be investigated. It is of note that the distribution of headache exacerbations in TTH seems to be different than migraine. In most previous studies, migraine attacks were reported to occur more often in the morning [1,13,14] and sometimes during sleep [15], although a few studies showed peak attack frequency in the afternoon [16].

There are some limitations to the present study. Firstly, external clock time was used for the time axis in this study. This approach might cause between-individual variations in the effect of time, which might disappear with consideration of individual sleep-wake cycles. However, whether to use external clock time or individual sleep-wake cycles as the time axis is dependent on the hypothesis. Second, computerized EMA is not perfect, although it has an advantage over recalled self-reporting because it is able to capture the time course of headache intensity in a more detailed and accurate manner. Signal-contingent recordings were not continuous, but were sparse so that brief changes in headache intensity might have been missed, which was partly solved by dividing time into specific time blocks, sacrificing high time resolution. In addition, although correspondence between momentary headache intensity and recording time was accurate, there might have been a lag between the time of headache exacerbation and the time of making an event-contingent recording, despite instruction to make event-contingent recordings as soon as possible when headache was acutely exacerbated. Third, although we tried to explore the effect of medication on the diurnal pattern of headache by analyzing the interaction with medication use, it would be better if we could investigate only patients who were free of medication, especially for the investigation of pathophysiology. Fourth, the number of patients was rather small, thus the generalizability of this study might be limited.

## Conclusions

Using computerized EMA, it was determined that there was a specific diurnal pattern of momentary headache intensity and the probability of acute headache exacerbations in TTH patients. These findings would be utilized for patient education and headache management.

## Abbreviations

TTH, Tension-type headache; EMA, Ecological momentary assessment; VAS, Visual analog scale; AIC, Akaike’s Information Criterion.

## Competing interests

The authors declare that they have no competing interests.

## Authors’ contributions

HK designed the study, collected the data, analyzed the data, performed the statistical analysis, interpreted the results, and drafted the manuscript. KY designed the study, interpreted the results, and drafted the manuscript. YY, GK and AA helped interpret the results and draft the manuscript. All authors read and approved the final manuscript.
